# The burden of headache disorders in Benin: national estimates from a population-based door-to-door survey

**DOI:** 10.1186/s10194-025-01992-7

**Published:** 2025-03-17

**Authors:** Thierry Adoukonou, Mendinatou Agbetou, Eric Dettin, Oyéné Kossi, Andreas Husøy, Dismand Houinato, Timothy J Steiner

**Affiliations:** 1 Department of Neurology, University of Parakou, Parakou, Benin; 2 Department of Neuromedicine and Movement Science, Norwegian University of Science and Technology (NTNU), Edvard Griegs gate, Trondheim, Norway; 3 Department of Neurology, University of Copenhagen, Copenhagen, Denmark; 4 Division of Brain Sciences, Imperial College London, London, UK

**Keywords:** Headache disorders, Migraine, Tension-type headache, Medication-overuse headache, Epidemiology, Burden of disease, Population-based survey, Health-care needs assessment, sub-Saharan Africa, Benin, Global Campaign against Headache

## Abstract

**Background:**

Continuing the series of population-based studies conducted within the Global Campaign against Headache, here we report estimates of headache-attributed burden among adults in Benin, West sub-Saharan Africa, adding to those already published of prevalence.

**Methods:**

In a cross-sectional survey using cluster-randomized sampling, we visited households unannounced in three geographical regions of Benin: Borgou, Atlantique and Littoral. We randomly selected and interviewed one adult member (18–65 years) of each household, using the HARDSHIP structured questionnaire. Screening and diagnostic questions based on ICHD-3 were followed by burden enquiry in multiple domains including symptom burden and impaired participation. Enquiry timeframes were 1 year, 3 months, 1 month and 1 day (headache yesterday). Data collection took place from May to July 2020.

**Results:**

There were 2,400 participants. Those reporting any headache spent, on average, 8.0% of their total time with headache of moderate-to-severe intensity. Females had more frequent headache than males. Participants with migraine spent twice as much time with headache as those with TTH (5.2% vs. 2.6%). Those with probable medication-overuse headache or other headache on ≥ 15 days/month spent over 50% of their time with headache. Factoring in prevalence and adjusting for age and gender, we estimated that 6.4–6.5% of all time among the adult population of Benin was spent with headache. An estimated 26.7% of the population were assessed as in need of (likely to benefit from) health care for headache.

**Conclusion:**

The burden of headache in Benin is substantial in terms of lost health. These findings are important to national health and economic policies.

## Background

Continuing the series of population-based studies conducted within the Global Campaign against Headache [1–8], here we report estimates of headache-attributed burden in Benin, West sub-Saharan Africa (SSA). These add to our previously published estimates of prevalence [8]. The data for the two studies were collected at the same time from the same participants.

Globally, headache disorders are prevalent and associated with high symptom burden [9–11], impaired participation in daily life, detriments to productivity and economic losses [10–13]. These may vary, dependent on factors such as geography, climate, culture, ethnicity and wealth, so country-based data are important to the formulation of health policy and allocation of health resources, particularly in countries where resources are scarce. The Global Campaign has focused recently on countries in western and central SSA [8, 14–16], where knowledge of headache was lacking. Among these countries, Benin is one of the world’s poorest [17], with a health-care system that is underdeveloped; life expectancy is under 60 years, and death rates for children under five years are among the world’s highest [18].

The study was of the headache disorders of public-health importance: migraine, tension-type headache (TTH), probable medication-overuse headache (pMOH) and other headache on ≥ 15 days/month (other H15+). The last was not further classified.

## Methods

We used the standardized methodology and questionnaire developed by the Global Campaign [19, 20] and already published in detail [8]. Here the methods are summarized.

### Ethics

The study was conducted in accordance with the Declaration of Helsinki [21] and with approvals from the Local Ethics Committee for Biomedical Research of the University of Parakou (CLERB-UP) (number 0168/CLERB-UP/P/SP/SA) and from academic and administrative authorities.

All participants gave verbal consent. Data were collected and managed in accordance with data-protection laws.

### Study design and procedures

A cross-sectional questionnaire-based survey of adults was conducted between May and July 2020. The three geographical regions of Borgou, Atlantique and Littoral were sampled in order to obtain a representative sample of the general population of Benin. Households in each region, and one adult member (aged 18–65 years) of each household (visited unannounced), were randomly selected.

Participants were interviewed using the structured Headache-Attributed Restriction, Disability, Social Handicap and Impaired Participation (HARDSHIP) questionnaire [20], translated into Central African French according to LTB’s translation protocol for lay documents [22]. Enquiry into headache-attributed burden encompassed symptom burden, expressed as frequency and usual intensity and duration of headache, impaired participation in paid work, household work and social or leisure activities (using the Headache-Attributed Lost Time [HALT] questionnaire [23]), willingness to pay (WTP) for effective health care for headache, and impact on quality of life (QoL) (using WHOQoL-8 [24]). Participants were asked to consider only the most bothersome headache type when more than one type was reported. Additional enquiries related to headache on the preceding day (“headache yesterday” [HY]).

Diagnoses were made algorithmically [20] according to ICHD-3 criteria [25], as previously reported [8].

Full details of these methods are available in our earlier publication [8].

### Statistics and analysis

Usual headache intensity was recorded as “not bad”, “quite bad” or “very bad”; these responses were converted to a numerical scale of 1–3, treated as continuous. Headache frequency in days/month and usual headache duration in hours were recorded as continuous data. Time spent in the ictal state (TIS) was determined at individual level as the product of headache frequency and duration (capped at 24 h to avoid overestimation, since frequency was reported as days not attacks per month), and expressed as the proportion of total time (pTIS = TIS/[30*24]). Headache-attributed lost health (i.e., diminution from perfect health) was computed at individual level as pTIS*DW, where DW (on a scale of 0–1) was the disability weight for the ictal state applied in the Global Burden of Disease study [26] for the specific headache type.

Impaired participation was quantified in lost complete days over the preceding 3 months, separately from paid work, household work and social or leisure activities (the first two considered to represent lost productivity). Using the accepted HALT methodology, days with less than half or nothing achieved were counted as whole days lost, and, in counterbalance, days with more than half or everything achieved were counted as no days lost [23]. In this analysis, we applied a value of zero to missing data since participants would not respond when the question was irrelevant (for example, unemployed participants could not lose days from paid work). Overall impaired participation with HY was counted as 0% on days when less than half or nothing had reportedly been achieved and as 100% when more than half or everything had been achieved.

Population-level estimates were derived by factoring in our prevalence estimates (from [8]) and correcting for age and gender.

WTP, per month, was recorded in West African francs (XOF) (at the time of the study, USD 1 = XOF 583). QoL was assessed as summed WHOQoL-8 scores on a scale of 8–40 [24], with higher scores indicating better QoL, and treated as continuous data.

A health-care needs assessment estimated the proportion of the population expected to benefit from effective professional headache care (the definition of “need”). We used the following criteria to identify those likely to benefit:

1. all those with H15+ (pMOH or other);
2. all those with migraine AND one or more of the following:
  - headache frequency ≥ 3 days/month;
  - moderate or severe headache intensity AND pTIS > 3.3%;
  - reporting ≥ 3 days/3 months lost from paid or household work;
3. all those with TTH AND one or more of the following:
  - moderate or severe headache intensity AND pTIS > 3.3%;
  - reporting ≥ 3 days/3 months lost from paid or household work.

We used means, standard errors of the mean (SEMs) and medians to report continuous variables. We used ANOVA for continuous data and chi-squared tests for categorical data to test for significance (set at *p* < 0.05).

Statistical analyses were conducted using SPSS version 28 for statistical analysis (SPSS, INC, Chicago, IL) and Microsoft Excel Version 16.

## Results

Our sample consisted of 2,400 participants, slightly younger (mean 32.1 years) than the general population of Benin aged 18–65 years (34.6 years; *p* < 0.001), with matching male-female ratio (51.3% vs. 49.8% male; *p* = 0.15) but less urbanized (30% vs. 48%; *p* < 0.001). Prevalence estimates adjusted for age and gender, already published [8] but repeated here because they are needed for population-level burden estimates, were 72.9% for all headache, 21.2% for migraine, 43.1% for TTH, 4.5% for pMOH and 3.1% for other H15+.

**Table 1:** Symptom burden and lost health, overall and by gender, for all headache and each headache type

	Overall	Male	Female	Male vs. female
	Mean±SEM, median			
**Frequency** (days/month)				
All headache	4.3±0.2, 2.0	3.6±0.2, 2.0	4.9±0.3, 2.0	**F(1**,** 1794) = 15.2**, *p*** < 0.001**
pMOH	24.1±0.7, 30.0	23.3±1.2, 26.0	24.4±0.8, 30.0	F(1, 99) = 0.6, *p* = 0.44
Other H15+	21.9±1.1, 22.0	21.0±1.6, 20.5	22.9±1.3, 22.5	F(1, 66) = 0.8, *p* = 0.37
Migraine	2.3±0.1, 2.0	2.1±0.1, 2.0	2.6±0.1, 2.0	**F(1**,** 562) = 5.0**, *p*** = 0.03**
TTH	2.3±0.1, 2.0	2.2±0.1, 2.0	2.5±0.1, 2.0	**F(1**,**1045) = 4.3**, *p*** = 0.04**
**Duration** (hours)				
All headache	19.3±1.0, 4.0	18.9±1.4, 3.0	19.7±1.3, 6.0	F(1, 1794) = 0.2, *p* = 0.7
pMOH	16.9±1.1, 24.0	16.9±1.7, 24.0	16.8±1.5, 24.0	F(1, 99) = 0.0, *p* = 0.97
Other H15+	46.8±16.6, 24.0	52.8±24.7, 24.0	40.0±22.0, 24.0	F(1, 66) = 0.1, *p* = 0.70
Migraine	27.7±1.4, 24.0	27.4±2.1, 24.0	27.9±1.9, 24.0	F(1, 562) = 0.0, *p* = 0.85
TTH	13.3±0.9, 2.0	12.6±1.2, 2.0	13.9±1.3, 2.0	F(1, 1045) = 0.5, *p* = 0.47
**Intensity** (not bad-quite bad-very bad, equated to 1, 2, 3 and treated as continuous data**)**				
All headache	41-906-849 (mean = 2.5)	22-471-397 (mean = 2.4)	19-435-452 (mean = 2.5)	*X*^*2*^(2, *N* = 1796) = 5.1, *p* = 0.08
pMOH	0-28-78 (mean = 2.7)	0-12-19 (mean = 2.6)	0-16-54 (mean = 2.8)	*X*^*2*^(2, *N* = 101) = 2.7, *p* = 0.15
Other H15+	0-31-37 (mean = 2.5)	0-21-15 (mean = 2.4)	0-10-22 (mean = 2.7)	***X***^***2***^**(2**, *N*** = 68) = 5.0**, *p*** = 0.03**
Migraine	2-135-427 (mean = 2.8)	0-57-216 (mean = 2.8)	2-78-211 (mean = 2.7)	*X*^*2*^(2, *N* = 564) = 4.8, *p* = 0.09
TTH	39-709-299 (mean = 2.3)	22-380-144 (mean = 2.2)	17-329-155 (mean = 2.3)	*X*^*2*^(2, *N* = 1047) = 2.8, *p* = 0.25
**Proportion of time in ictal state (pTIS)** (%)				
All headache	8.0±0.4, 0.8	6.4±0.6, 0.8	9.6±0.7, 1.1	**F(1**,** 1794) = 12.7**, *p*** < 0.001**
pMOH	52.1±3.5, 50.0	50.7±6.1, 50.0	52.7±4.3, 53.3	F(1, 99) = 0.1, *p* = 0.79
Other H15+	51.0±4.8, 50.0	44.8±6.7, 50.0	58.0±6.7, 56.7	F(1, 66) = 1.9, *p* = 0.17
Migraine	5.2±0.3, 2.7	4.7±0.4, 2.1	5.6±0.4, 3.3	F(1, 562) = 2.8, *p* = 0.10
TTH	2.6±0.2, 0.4	2.2±0.2, 0.4	3.0±0.3, 0.5	F(1, 1045) = 3.8, *p* = 0.05
**Lost health** (%)				
Migraine	2.3±0.1, 1.2	2.1±0.2, 0.9	2.5±0.2, 1.4	F(1, 562) = 2.8, *p* = 0.10
TTH	0.1±0.0, 0.0	0.1±0.0, 0.0	0.1±0.0, 0.0	F(1, 1045) = 3.8, *p* = 0.05

pMOH: probable medication-overuse headache; H15+: headache on ≥ 15 days/month; TTH: tension type headache; p-values indicating significance (< 0.05) are emboldened

**Table 2:** Impaired participation in the last 3 months, overall and by gender, for all headache and each headache type

	Overall	Male	Female	Male vs. female
	mean±SEM, median			
**Lost days from paid work (HALT questions 1 + 2)**				
All headache	0.4±0.0, 0.0	0.4±0.0, 0.0	0.5±0.1, 0.0	F(1, 1795) = 0.8, *p* = 0.40
pMOH	1.8±0.4, 0.0	1.7±0.6, 0.0	1.9±0.6, 0.0	F(1, 99) = 0.0, *p* = 0.84
Other H15+	1.4±0.4, 0.0	1.5±0.6, 0.0	1.3±0.5, 0.0	F(1, 66) = 0.0, *p* = 0.87
Migraine	0.5±0.1, 0.0	0.6±0.1, 0.0	0.5±0.1, 0.0	F(1, 562) = 0.7, *p* = 0.42
TTH	0.2±0.0, 0.0	0.2±0.0, 0.0	0.2±0.0, 0.0	F(1, 1045) = 0.3, *p* = 0.59
	**F(3, 1776) = 51.7, ** ***p*** ** < 0.001**			
**Lost days from household work (HALT questions 3 + 4)**				
All headache	0.2±0.0, 0.0	0.2±0.0, 0.0	0.3±0.0, 0.0	F(1, 1795) = 3.1, *p* = 0.08
pMOH	1.5±0.3, 0.0	1.3±0.6, 0.0	1.7±0.4, 0.0	F(1, 99) = 0.2, *p* = 0.63
Other H15+	0.4±0.2, 0.0	0.3±0.2, 0.0	0.7±0.3, 0.0	F(1, 66) = 1.4, *p* = 0.23
Migraine	0.2±0.0, 0.0	0.2±0.1, 0.0	0.2±0.0, 0.0	F(1, 562) = 0.1, *p* = 0.79
TTH	0.1±0.0, 0.0	0.1±0.0, 0.0	0.1±0.0, 0.0	F(1, 1045) = 0.0, *p* = 0.84
	**F(3, 1776) = 61.5, ** ***p*** ** < 0.001**			
**Lost days from social or leisure activities (HALT question 5)**				
All headache	0.0±0.0, 0.0	0.0±0.0, 0.0	0.0±0.0, 0.0	F(1, 1795) = 2.3, *p* = 0.13
pMOH	0.1±0.1, 0.0	0.1±0.1, 0.0	0.1±0.1, 0.0	F(1, 99) = 0.2, *p* = 0.64
Other H15+	0.0±0.0, 0.0	0.0±0.0, 0.0	0.0±0.0, 0.0	F(1, 66) = 0.0, *p* = 0.93
Migraine	0.0±0.0, 0.0	0.0±0.0, 0.0	0.0±0.0, 0.0	F(1, 562) = 2.0, *p* = 0.16
TTH	0.0±0.0, 0.0	0.0±0.0, 0.0	0.0±0.0, 0.0	F(1, 1045) = 2.4, *p* = 0.13
	**F(3, 1776) = 6.3, ** ***p*** ** < 0.001**			

HALT: Headache-Attributed Lost Time (index); pMOH: probable medication-overuse headache; H15+: headache on ≥ 15 days/month; TTH: tension-type headache; p-values indicating significance (< 0.05) are emboldened

### Symptom burden

For all headache, mean frequency was 4.3 days/month and mean duration was 19.3 h. Medians evidenced skewed data (Table 1). Mean intensity was 2.5 (moderate-to-severe). Frequency of headache was higher among females than males (4.9 vs. 3.6 days/month; *p* < 0.001), but duration and intensity were similar between genders. Mean individual pTIS was 8.0%.

For migraine, mean headache frequency was 2.3 days/month, higher among females (2.6 days/month) than males (2.1 days/month; *p* = 0.03). Mean intensity of headache (2.5: moderate-to-severe), headache duration (27.7 h) and pTIS (5.2%) were not significantly different between genders (Table 1). With DW = 0.441 [26], individual migraine-attributed lost health was 2.3%, similar in males and females.

For TTH, mean frequency was 2.3 days/month, higher among females (2.5 days/month) than males (2.2 days/month; *p* = 0.04) (Table 1). Mean intensity (2.3: moderate) and duration (13.3 h) did not differ between genders, but pTIS (overall 2.6%) was higher among females than males (3.0% vs. 2.2%; *p* = 0.05). With DW = 0.037 [26], individual lost health was only 0.1%.

**Fig. 1:**
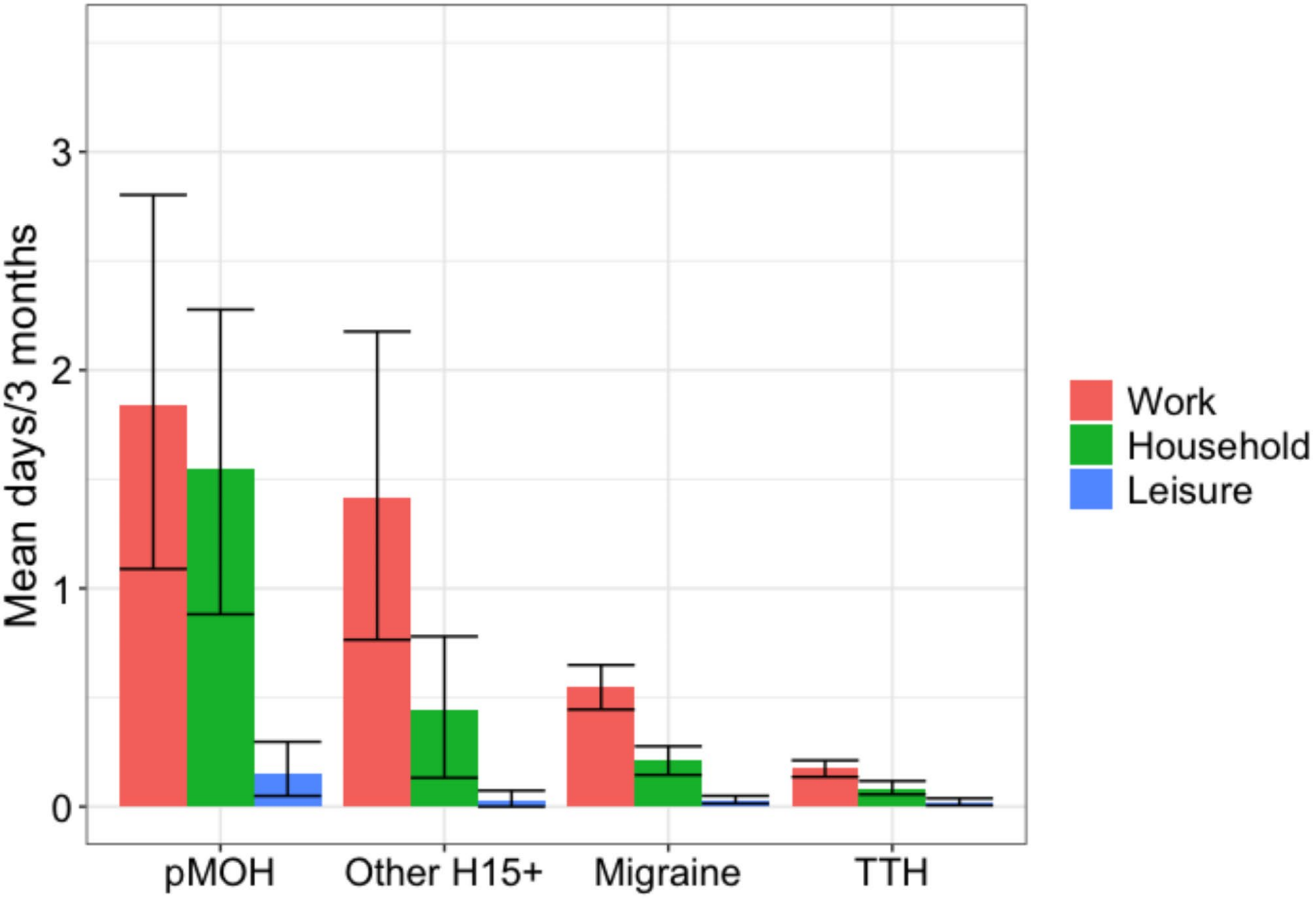
Impaired participation in paid (red) and household work (green) and in social or leisure activities (blue) by headache type. Error bars are 95% CIs. pMOH: probable medication-overuse headache; H15+: headache on ≥ 15 days/month; TTH: tension-type headache

Frequency of pMOH was 24.1 days/month, of other H15+ 21.9 days/month. Participants with either spent, on average, more than half of their time with headache (pMOH 52.1%; other H15+ 51.0% [both with duration capped at 24 h]) (Table 1). Females with other H15+ reported higher headache intensity (mean 2.7) than males (mean 2.4; *p* = 0.03).

### Impaired participation

For all headache, average lost days from paid work, household work and social or leisure activities were 0.4, 0.2 and 0.0 per 3 months respectively (Table 2). Lost days from paid and household work were 0.5 and 0.2 per 3 months among those with migraine, 0.2 and 0.1 among those with TTH, 1.8 and 1.5 among those with pMOH, and 1.4 and 0.4 days among those with other H15+ (Table 2). Across all headache types, these differences were significant (*p* < 0.001; also shown in Fig. 1). Only those with pMOH had a detectable loss in days from social or leisure activities (0.1 days/3 months). There were no significant gender-related differences (Table 2).

### Headache yesterday

Among *n* = 354 reporting HY, its mean duration was 10.0 h and mean intensity 2.4 (moderate-to-severe) (Table 3).

With regard to impaired participation with HY, 28.7% reported doing everything as normal, 19.7% more than half, 22.5% less than half and 29.2% nothing (Table 3). Applying the accepted HALT methodology of interpreting more than half as 100% and less than half as 0% [23], we calculated overall impaired participation of 51.7% among those with HY. There were no gender-related differences.

### Quality of life and willingness to pay

Participants with headache of any kind reported lower QoL than those without headache (28.9). Differences across all headache types and no headache were significant (*p* < 0.001), although the diminutions in QoL were appreciable only for pMOH (27.4) and other H15+ (26.8) (Table 4).

On average, those with pMOH were willing to pay XOF 2,205/month for effective treatment, those with migraine XOF 1,516/month, those with TTH XOF 1,461/month and those with other H15+ XOF 1,302/month (Table 4). At the time of the study, these were in the range US$ 2.20–3.80. The differences between headache types were not significant.

**Table 3:** Duration, intensity and impaired participation associated with headache yesterday, overall and by gender

	Overall	Male	Female	Male vs. female
**Duration** (hours) (mean±SEM, median)	10.0±0.5, 4.0	8.8± + 0.8, 4.0	10.8±0.7, 5.0	F(1, 352) = 3.6, *p* = 0.06
**Intensity** (n [%])				*X*^*2*^(2, *N* = 356) = 3.7, *p* = 0.16
1 (not bad)	1 (0.3)	1 (0.7)	0 (0.0)	
2 (quite bad)	202 (56.7)	90 (61.2)	112 (53.6)	
3 (very bad)	153 (43.0)	56 (38.1)	97 (46.4)	
mean*	2.4	2.4	2.5	
**What done** (n [%])				*X*^*2*^(3, *N* = 356) = 3.6, *p* = 0.30
everything	102 (28.7)	37 (25.2)	65 (31.1)	
more than half	70 (19.7)	28 (19.0)	42 (20.1)	
less than half	80 (22.5)	40 (27.2)	40 (19.1)	
nothing	104 (29.2)	42 (28.6)	62 (29.7)	

*treating as continuous data; there are no p-values indicating significance (< 0.05)

**Table 4:** Willingness to pay (WTP) for effective treatment, and quality of life measured with WHOQoL-8 [24], overall and by headache type

Willingness to pay (XOF*) (mean±SEM; median)	
pMOH	2,205±275; 1,000
Other H15+	1,302±240; 700
migraine	1,516±100; 700
TTH	1,461±104; 700
	F(3, 1531) = 2.2, *p* = 0.09
**Quality of life** (scale 8–40) (mean±SEM; median)	
pMOH	27.4±0.4, 28.0
Other H15+	26.8±0.6, 28.0
migraine	28.2±0.2, 28.0
TTH	28.3±0.1, 28.0
no headache	28.9±0.1, 29.0
	**F(4, 2378) = 9.7, ***p* < 0.001

*At the time of the study, USD 1 = XOF 583; pMOH: probable medication-overuse headache; H15+: headache on ≥ 15 days/month; TTH: tension-type headache; p-values indicating significance (< 0.05) are emboldened

### Population-level estimates

For all headache, population-level pTIS was estimated from mean headache frequency, mean usual duration capped at 24 h and 1-year prevalence (from [8]), adjusting for age and gender, to be 6.4%. Most time was spent with pMOH (2.5%), followed by other H15+ (1.6%), migraine (1.2%) and TTH (1.1%).

For all headache, almost the exact same estimate of pTIS (6.5%) was obtained from 1-day prevalence and mean duration of HY, also adjusting for age and gender.

Headache-attributed impaired participation at population-level, according to HALT data, was estimated as 0.3 days lost during the preceding 3 months from paid work and 0.2 days lost from household work, these constituting lost productivity (Table 5). Migraine, TTH and pMOH contributed equally to the losses from paid work (0.1 days/3 months), whereas pMOH was the only measurable cause of lost household work (Table 5). There were no reported losses from social or leisure activities.

**Table 5:** Proportion of time in ictal state and impaired participation at population level, by headache type and by timeframe of enquiry (adjusted for age and gender)

Headache type	Estimated pTIS (%)		Estimated impaired participation			
	According to 1-year prevalence and reported average frequency and usual duration	According to prevalence and duration of headache yesterday	According to HALT data (lost days/3 months)			According to headache yesterday
			**Lost productivity**		**Social or leisure activities**	**Total impaired participation** **(%)**
			**Paid work**	**Household work**		
Any headache	6.4	6.5	0.3	0.2	0.0	8.2
pMOH	2.5		0.1	0.1	0.0	
Other H15+	1.6		0.0	0.0	0.0	
Migraine	1.2		0.1	0.0	0.0	
TTH	1.1		0.1	0.0	0.0	

pTIS: proportion of time in ictal state; HALT: headache-attributed lost time; pMOH: probable medication overuse headache; H15+: headache on ≥ 15 days/month; TTH: tension type headache

### Health-care needs assessment

A total of 630 (26.3%) participants fulfilled one or more of our criteria for health-care need (Table 6). Adjusted for age and gender, this equated to 26.7%. The majority had migraine (12.0%), followed by rather similar proportions with H15+ (7.6%) or TTH (7.1%). Few participants (2.5%) were considered to be in need of headache care because of lost productivity (criteria 4 and 6: Table 6).

**Table 6:** Health-care needs assessment

Criterion fulfilled		Proportion of sample		Estimated proportion of adult population*
		*n*	%	% [95% CI]
1	Headache on ≥ 15 days/month	169	7.0	7.6 [6.6–8.8]
2	Migraine on ≥ 3 days/month	199	8.3	8.4 [7.3–9.6]
3	Migraine and pTIS > 3.3% and moderate-to-severe intensity	216^1^	9.0	9.0 [7.9–10.2]
4	Migraine and ≥ 3 lost days from paid and/or household work per 3 months	36^2^	1.5	1.4 [1.0–2.0]
5	TTH and pTIS > 3.3% and moderate-to-severe intensity	155	6.5	6.4 [5.5–7.4]
6	TTH and ≥ 3 lost days from paid and/or household work per 3 months	24^3^	1.0	1.0 [0.7–1.5]
One or more of criteria 1–6		630	26.3	26.7 [24.9–28.5]

*Age- and gender-corrected; ^1^of whom 136 also fulfilled criterion 2; ^2^of whom 25 also fulfilled criterion 2, 19 also fulfilled criterion 3, and 18 also fulfilled criteria 2 and 3; ^3^of whom 7 also fulfilled criterion 5; pTIS: proportion of time in ictal state; TTH: tension type headache

## Discussion

This is the first population-based study utilizing the standardized methodology developed by the Global Campaign against Headache [19, 20] to describe the burden of headache disorders in a country in West sub-Saharan Africa (SSA). This methodology was developed by expert consensus, primarily to improve the quality of population-based studies of headache disorders [19], but it was also intended that the Global Campaign would adopt it across its entire series of studies. This key attribute of these studies greatly facilitates comparisons between them.

Our previous study found headache to be a near-universal experience in Benin, with a lifetime prevalence of 95.2% [8]. Consequently, it is unsurprising that symptom burden at population level is high in this country: 6.4% of all adult time in Benin is spent with headache of, on average, moderate-to-severe intensity. Two thirds of this is attributed to H15+. Nevertheless, we estimated relatively little lost productivity at population level (0.3 lost days/3 months, on average, from paid work and 0.2 lost days/3 months from household work), which might be expected in an economy in which 90% of the workforce was employed in the informal sector [27]. At individual level, some participants were clearly seriously affected by headache. Those with H15+ spent, on average, more than half of their time with headache, and, presumably consequentially, reported the lowest QoL. Lost days from paid work were 1.8 days/3 months among those with pMOH and 1.4 days/3 months among those with other H15+.

Estimates of this sort are subject to the uncertainties in recall over 3 months. To counter these, we also made estimates based on HY, likely to be free from recall error [28], finding a confirmatory 6.5% of all adult time in Benin to be spent with headache. We therefore have confidence in these estimates of pTIS.

Yet, there were apparent discrepancies. Recalled mean headache duration was 19.3 h, whereas mean duration of HY was 10.0 h. An incomplete explanation of this apparent discrepancy is that duration of HY was, obviously, capped at 24 h; when usual headache duration is > 24 h, HY cannot wholly capture it. But the two methods of estimating population-level pTIS produced almost identical findings (6.4% and 6.5%), counterintuitively, when mean recalled headache duration was nearly double mean duration of HY. In the calculation of pTIS from frequency and usual headache duration, the latter was also capped at 24 h, since frequency was reported as days/month rather than attacks/months. Additionally, there was evidence that participants underestimated headache frequency (observed 1-day prevalence was higher [14.8%] than predicted from 1-year prevalence and mean headache days/month [10.7%] [8]).

A less easily explained discrepancy was in the two measures of impaired participation. While, according to HALT data and 3 months’ recall, only 0.3 days/3 months were lost from paid work and 0.2 days/3 months from household work, overall impaired participation with HY, in stark contrast, was 8.2%. The former estimate was among all those reporting any headache in the preceding year (*n* = 1,797), the latter among the much smaller – although still considerable – number reporting HY (*n* = 354). We can calculate percentage losses from HY data because these provide denominators, since the enquiry pertained to whatever had been planned for one specific day. We cannot reliably do so from HALT data because the denominators (numbers of days set aside for paid or household work per 3 months by each participant) were unknown. However, 0.3 days lost from 65 days (a 5-day working week) would be only 0.5%. This strongly suggests that impaired participation in daily activities, including lost productivity, was substantially underestimated in the recalled data of HALT. As noted in Methods, we conservatively applied a value of zero to missing HALT data. We did this to preserve the denominator, and on the basis that participants would not respond to questions deemed irrelevant (for example, unemployed participants could not lose days from paid work). We comment further on this below.

The enquiry into HY (“How did the headache you had yesterday affect your ability to do your day-to-day activities [all the things that you would normally have done]?”) should have captured losses from social or leisure activities as well as losses from paid and household work. However, it is striking that recalled losses over 3 months from social or leisure activities (HALT data) were unmeasurably low (Table 5). There is more than one way of interpreting this finding, with no means of knowing the truth: that such losses were not well remembered; that there was disinclination to report them; that headache was not allowed to disrupt social or leisure activities; that little time in Benin was given to social or leisure activities. Either of the first two interpretations might partially explain the discrepancy between HALT and HY estimates of impaired participation.

Females in Benin spent significantly more time with headache than did males. More precisely, females with migraine or TTH reported more headache days than males, whereas duration and intensity of headaches were the same. We have previously shown, for migraine, that lost productivity correlates well with frequency but not with duration, and only to a lesser degree with intensity [29], but, in the present analyses, higher frequency among females did not translate into greater losses from productivity. Any attempt to explain this in terms of gender-related differences in productive activity, or by attributing greater resilience to females, would be entirely speculative.

Quality of life was impacted by headache, especially by pMOH and other H15+, as might be expected. However, it is notable that those with no headache reported an average score of only 28.9/40. Willingness to pay for effective care was greatest (in terms of XOF/month) for pMOH, ahead of migraine, but the WTP range was low: less than the equivalent of US$ 4/month. WTP is a good measure conceptually, since it should take account of all negative aspects of having headache, including those that are otherwise unmeasurable. But it is highly influenced by ability to pay. As noted above, Benin is among the world’s poorest countries [17].

Those with H15+ (7.6% of adults, almost 60% of these with pMOH) might deserve special attention in the provision of health care. MOH, in theory, is relatively easy to treat – essentially by supported medication withdrawal; further, also in theory, it is entirely preventable [30, 31]. But prevention and effective management both call for knowledge and understanding. The potential for health gain among these people alone, from headache care made accessible through the implementation of structured headache services supported by education of health-care providers and the general public [32], is substantial. This brings us to our health-care needs assessment.

According to our criteria, over a quarter (26.7%) of Benin’s population aged 18–65 years would be expected to benefit from effective and accessible headache care. Despite the individual burden attributable to H15+, the majority of these had migraine. We believe it to be uncontroversial to assess all those with H15+ (whether pMOH or other) as in need of professional care, since any degree of recovery is unlikely without it. Table 6 shows that most of those with migraine and assessed as needing care reported frequencies of ≥ 3 days/month, often considered to be the threshold for preventative medication [33–37], therefore requiring professional care. Those with migraine or TTH and ≥ 3 lost days from paid and/or household work per 3 months also appear to be obvious candidates for professional care. These groups total 16.5%, whose needs were very clear (Table 6), while our other criteria, which bring in almost 10% more, might be considered more questionable in a resource-limited country.

Finally, we make brief comparisons with our similar studies in nearby Mali (to the north) [16] and Cameroon (to the south-east) [14, 15]. In Mali, a low-income country, headache reportedly affected 90.8% of the adult population with a mean frequency of 3.5 days/month. At population level, 3.6–5.8% of all time was spent with headache according to the two methods of measurement, which led to 1.2 and 0.9 days/3 months lost from paid and household work, considerably more than reported in Benin. But overall impaired participation according to HY data was 3.6% in Mali [16], less than half the 8.2% in Benin. The prevalences of H15+ were very different (age- and gender-adjusted: 4.5% in Mali, 7.6% in Benin), which might have distorted how these estimates related to one another. Almost a quarter (23.4%) of Mali’s adult population were estimated to need headache care [16], a proportion similar to Benin’s 26.7%. In Cameroon, a lower-middle income country, headache reportedly affected 76.4% of the adult population [14] with a higher mean frequency of 6.7 days/month [15]. At population level, 6.1–7.4% of all time was spent with headache, mostly attributed to H15+ (5.3% of all time) with its very high prevalence of 13.1% [15]. Mean lost days/3 months were 2.5 from paid work and 2.2 from household work, higher than in Mali and much higher than in Benin. Overall impaired participation was 6.9% according to HY data [15], higher than in Mali but lower than in Benin. Again, the differences in H15+ might have distorted how estimates related to one another. An estimated 37.0% of adults in Cameroon needed headache care [15], a proportion much expanded by those with H15+.

### Strengths and limitations

We used standardized and well-tested methodology [19, 20], with ICHD-based diagnoses. The HARDSHIP questionnaire [20] had been used previously, in the same translation, in Cameroon [14, 15]. The sample was drawn from multiple regions in order to be representative of Benin’s diverse population [8], and, with *N* = 2,400, exceeded the recommended *N* > 2,000 [19]. The participating proportion was a high 94.1% [8]. These were strengths. The sample (slightly younger, and less urbanized) did not perfectly match the general population of Benin aged 18–65 years, but adjustments were made for the former in the prevalence estimates [8]. The limitation of reliance on participants’ recall, as in all retrospective observational studies, was mitigated by our collection of data on HY. Applying a value of zero to missing HALT (impaired participation) data may have been over-conservative. Further, if unemployment was actually due to headache, this potentially heavy element of headache-attributed burden would have been missed unless the participant recorded 90 days lost (rather than making no response). A general limitation of cross-sectional studies with a single encounter is inability to diagnose H15+ beyond the recognition of association with acute medication overuse (pMOH). Our focus on the most bothersome headache, with only one diagnosis per participant when more than one type was reported, probably led to underestimated TTH-attributed burden.

## Conclusions

With headache a near-universal experience in Benin, and 6.4% of all adult time in Benin spent with headache of moderate-to-severe intensity, the negative impact of headache on population health in Benin is substantial. At individual level, many are seriously affected, not least the 7.6% with H15+, who reported headache for more than half of their time. On our assessment, 16.5% of adults in Benin have very clear need for headache-related health care (and a further 10% have less clear need). How important these findings are to national health policy is debatable in a resource-limited country with health-care priorities that are, obviously, very much higher. As for economic policy, estimated lost productivity was lower than might be expected, but relevant to this was that 90% of the workforce were employed in the informal sector.

## Data Availability

The original data are held at Department of Neurology, University of Parakou, Benin, and the analytical set at Norwegian University of Science and Technology, Trondheim, Norway. When analyses are completed, anonymised data will be available on request for academic purposes, in line with the policy of the Global Campaign against Headache.

## Abbreviations

ANOVA: analysis of variance
CI: confidence interval
d/m: days/month
DW: disability weight
GBD: Global Burden of Disease (study)
H15+: headache on ≥15 days/month
HALT: Headache-Attributed Lost Time (index)
HARDSHIP: Headache-Attributed Restriction, Disability, Social Handicap and Impaired Participation (questionnaire)
HY: headache yesterday
ICHD: International Classification of Headache Disorders
MOH: medication-overuse headache
pMOH: probable MOH
pTIS: proportion of time in ictal state
QoL: quality of life
SEM: standard error of the mean
SSA: sub-Saharan Africa
TIS: time in ictal state
TTH: tension-type headache
WHOQoL-8: World Health Organization quality of life (8-item questionnaire)
XOF: West African franc
